# The new era of precision population health: insights for the *All of Us* Research Program and beyond

**DOI:** 10.1186/s12967-018-1585-5

**Published:** 2018-07-27

**Authors:** Courtney R. Lyles, Mitchell R. Lunn, Juno Obedin-Maliver, Kirsten Bibbins-Domingo

**Affiliations:** 1 Center for Vulnerable Populations, University of California, San Francisco, CA USA; 2 Division of General Internal Medicine at Zuckerberg San Francisco General Hospital, Department of Medicine, University of California, San Francisco, CA USA; 3 Division of Nephrology, Department of Medicine, University of California, San Francisco, CA USA; 4 Department of Obstetrics, Gynecology & Gynecologic Subspecialties, University of California, San Francisco, CA USA; 5 Department of Epidemiology & Biostatistics, University of California, San Francisco, CA USA

**Keywords:** Precision medicine, Population health, Precision public health, Precision population health, Community engagement, Personalized medicine

## Abstract

Although precision medicine has made advances in individualized patient treatments, there needs to be continued attention on tailored population health and prevention strategies (often termed “precision population health”). As we continue to link datasets and use “big data” approaches in medicine, inclusion of diverse populations and a focus on disparities reduction are key components within a precision population health framework. Specific recommendations from the *All of Us* Research Program and the Precision Public Health Summit provide examples for moving this field forward.

## Background

Although early advancements in precision medicine have emphasized individualized genetic treatments, there are increasing calls for precision approaches to include social, behavioral, and environmental factors [1], alongside efforts to increase the overall diversity of participants in precision medicine initiatives. Reframing and broadening precision medicine beyond ‘omics’ (e.g., genomics, proteomics, metabolomics) has been referred to as “precision population health” or “precision public health.”

These goals of precision population health have been reflected in the US Precision Medicine Initiative, including the one-million-person research cohort called the *All of Us* Research Program (AoURP) [2]. With AoURP having just launched national recruitment, the US National Institutes of Health also recently funded AoU Community Engagement Partners to “educate, motivate, and facilitate enrollment… [to] reflect the diversity of the United States, including those historically underrepresented in biomedical research.” Community Engagement Partners are relevant in addressing potential challenges for the field of precision medicine. A limited number of underrepresented research participants can lead to inaccurate scientific conclusions, and increasing their numbers remains challenging in both clinical and genomics research [3].

In parallel with AoURP, the University of California, San Francisco; the Bill and Melinda Gates Foundation; and the White House Office of Science, Technology, and Policy convened a Precision Public Health Summit in 2016 [4] to tackle the challenges and opportunities of working in this multi-faceted space. Stakeholders in attendance included community members, government officials, business and industry leaders, academics, and community-based organizations interested in designing tailored public health programs, with a specific emphasis on child health during the first 1000 days of life.

We share here early lessons learned from planning for as well as leading an AoU Community Engagement Partner award for the sexual and gender minority (SGM) communities and convening stakeholders at the Precision Public Health Summit.

## Community engagement at the local level

The engagement needed to advance precision health goes further than diverse recruitment in research studies. Rather, local community members who are the primary beneficiaries of health programs should be true partners, and the AoU Community Engagement Partners represent a critical step toward this goal. Community members can add meaning and priority to the vast amount of precision data, guiding which types of data should be analyzed and combined (e.g., clinical data with publicly available datasets), as well as which issues or topics should be studied first. Furthermore, we will need ‘citizen scientists’ to submit their own data to our research and programmatic efforts—such as local information about environmental exposures or health behavior data. Participant-generated health data collection processes will never be successful unless stakeholders help to set the agenda and are assured that the information is not used to worsen the stigmatization or profiling of individuals/groups.

The vision for community engagement in precision population health includes engaging participants in setting research priorities and ensuring that they receive research findings in ways that are accessible, meaningful, and foster education and implementation (in the spirit of community-based participatory research). To make that vision more tangible, we heard concrete examples during the Precision Public Health Summit, such as the Connected Health Cities program in the UK [5], where community members actively vote on the acceptability of linking personal health data with other public datasets. Similarly, in our research activities that preceded our AoU Engagement Partner work, we used digital community forums to solicit thousands of research priorities for SGM people. We continue to give community members the opportunity to engage in discussions online, via social media, and through in-person events and community listening sessions. We are committed to supporting an open communication channel in perpetuity (and providing personalized, real-time answers).

## Broad data capture and data sharing

Data integration is not a new concept in precision medicine, as data linkage is a central mission of precision approaches. For example, the AoURP recognized the limited utility of genetic data without linked clinical, behavioral, and contextual data such as neighborhood and environmental factors. In its original protocol, AoURP includes questionnaires, electronic health record data, and physical measurements (height/weight, blood pressure) in addition to biological specimens (blood, urine) for ‘omic’ analyses. However, consensus is lacking about how much data we can reasonably collect or the specific data elements or measures that deserve priority (e.g., neighborhood food access vs. self-reported health behaviors). While programs like AoURP cannot wait to enroll participants before determining every data element of interest, we must not lose our focus on holistic data capture while improving the processes that will facilitate future data collection. Moreover, beyond participant-generated health data, we must actively consider using existing public datasets not usually considered health data (e.g., crime data, public social media posts) with the recognition that precision health necessitates entirely new data integration strategies.

Despite the desire and ability to more easily combine data, data alone are insufficient. There are multiple immediate needs to enhance data integration—from infrastructure needs to the governance structures that best engender trust between institutions and participants. At the Summit, we heard community-driven approaches for governance in the California Preterm Birth Initiative as a goal of their collective impact work [6], with specifics about investigating poor housing, transportation, and education as priorities. In our research with SGM people, communication about data collection, privacy, and security is paramount to building trust. We regularly update our data security processes to protect participants (e.g., data encryption at rest as well as during transmission, two-factor authentication) and actively communicate with our communities about what these new methods mean and how they work.

## Social justice through better collaborations

Finally, the commitment to diversify recruitment processes presents a unique opportunity to ensure that precision population health includes disparities reduction and social justice at its core. Because of the overwhelming evidence that poor health outcomes disproportionately burden specific communities, attention to the constellation of individual, social, environmental, and structural determinants of disparities in our communities is vital. Most of the previous major genomics-focused precision medicine projects have not had an explicit goal of disparities reduction; we see the progress from AoU Community Engagement Partners as the right moment to address this issue head on.

In order to maximize social justice, we need to break down silos between medicine, public health, and ‘omics’ science as well as institutional barriers between local/regional/national government departments, academia, healthcare systems, and industry. We learned concrete lessons at the Summit from an existing municipal and academic partnership in Cincinnati. This program overlaid pediatric asthma readmissions data from the electronic health record on top of public housing data, mobilizing medical–legal partnerships while patients were in the hospital to act on public housing violations to prevent future admissions [7]. This example demonstrates that precision approaches can act as both a root-cause analysis to discover trends, but also as an intervention strategy combining evidence-based medicine/public health and social policy. Our research mirrors this approach: first collecting data on SGM people that have been left out of almost all large-scale research studies to date, and then working to craft tailored interventions.

## Conclusion

We provided examples from on-the-ground work in precision population health—echoing decades of work in community engagement, data integration, and social justice—while highlighting priority areas to move this work forward. Precision approaches are rooted in the ability to collect more data, integrate more data types, and use enhanced computing power to uncover new insights about complex health issues. But we must not forget the parallel objectives within the Precision Medicine Initiative [8] and AoURP to develop new strategies and interventions using these collective data to improve prevention and treatment with the explicit goal of reducing health disparities.

Moving forward, we must continue to push for broader conceptualization of precision population health that can tailor health programs using individual and community level information. Building upon the early examples presented here, we see additional next steps to maximize the potential of precision population health efforts; many of these are being implemented in AoURP:

1. Work to raise awareness within the general public about their role in contributing data, identifying research priorities, informing implementation programs to ensure precision health approaches improve health in their communities.
2. Galvanize integrated healthcare systems to intervene on population health and social determinants concepts as core issues of precision population health, to realize the goal of “learning health systems” [9, 10].
3. Build infrastructure and leadership skills within public health departments (including training new professionals in precision population health) to advance data integration and community engagement efforts, guided by social justice as a core principle.
4. Share more lessons learned from the local level, so that communities working on similar issues can build upon and adapt the work of others.

We are inspired by our early experiences at the Precision Public Health Summit and SGM community engagement and research. We feel that broader application of these principles would help us to achieve substantial and rapid gains in population health, rather than waiting for individualized advancements in precision medicine to drive the progress.

## Abbreviations

AoURP: *All of Us* Research Program
SGM: sexual and gender minority
